# Integrating attention mechanism and boundary detection for building segmentation from remote sensing images

**DOI:** 10.3389/fnbot.2024.1482051

**Published:** 2025-01-14

**Authors:** Ping Liu, Yu Gao, Xiangtian Zheng, Hesong Wang, Yimeng Zhao, Xinru Wu, Zehao Lu, Zhichuan Yue, Yuting Xie, Shufeng Hao

**Affiliations:** ^1^College of Artificial Intelligence, Taiyuan University of Technology, Jinzhong, Shanxi, China; ^2^Business School of Northeast Normal University, Northeast Normal University, Changchun, Jilin, China; ^3^College of Mechanical and Vehicle Engineering, Taiyuan University of Technology, Jinzhong, Shanxi, China

**Keywords:** building segmentation, remote sensing images, convolutional neural, attention mechanism, boundary loss function

## Abstract

Accurate building segmentation has become critical in various fields such as urban management, urban planning, mapping, and navigation. With the increasing diversity in the number, size, and shape of buildings, convolutional neural networks have been used to segment and extract buildings from such images, resulting in increased efficiency and utilization of image features. We propose a building semantic segmentation method to improve the traditional Unet convolutional neural network by integrating attention mechanism and boundary detection. The attention mechanism module combines attention in the channel and spatial dimensions. The module captures image feature information in the channel dimension using a one-dimensional convolutional cross-channel method and automatically adjusts the cross-channel dimension using adaptive convolutional kernel size. Additionally, a weighted boundary loss function is designed to replace the traditional semantic segmentation cross-entropy loss to detect the boundary of a building. The loss function optimizes the extraction of building boundaries in backpropagation, ensuring the integrity of building boundary extraction in the shadow part. Experimental results show that the proposed model AMBDNet achieves high-performance metrics, including a recall rate of 0.9046, an IoU of 0.7797, and a pixel accuracy of 0.9140 on high-resolution remote sensing images, demonstrating its robustness and effectiveness in precise building segmentation. Experimental results further indicate that AMBDNet improves the single-class recall of buildings by 0.0322 and the single-class pixel accuracy by 0.0169 in the high-resolution remote sensing image recognition task.

## 1 Introduction

With the rapid development of the social economy, buildings have become an important part of the city. As the urban landform is constantly changing, building numbers, shapes, and sizes are increasingly diverse. Meanwhile, high-resolution images have more apparent features, richer texture information, and more prominent feature information in the image pixels (Liu et al., 2016). Thus, accurate extraction of buildings from high-resolution images is important for urban management, urban planning, mapping, and navigation.

The traditional methods of automatic building extraction can be divided into two categories based on edges and based on regions. Lin and Nevatia (1998) used the method of edge detection to determine the parallel relationship between lines and determine the rectangle to form the outline of the building to extract the geographical location of the building. Aytekın et al. (2012) combined spectral characteristics and spatial features to successfully extract buildings and roads from the complex background of satellite images. Izadi and Saeedi (2010) proposed a building detection method based on hierarchical multilayer features for image segmentation using color. Wegne et al. (2011) proposed a method to detect buildings with irregular shapes by combining optical and interferometric SAR (synthetic aperture radar) features.

With the rapid development of machine learning and deep learning, the mainstream building extraction method gradually shifts to pixel-level segmentation. Meedeniya et al. (2020) proposed a novel automated methodology based on learning models to identify land usage and coverage. The method integrates satellite imagery with Foursquare venue data to enhance the detail and quality of land-use visualization. Long et al. (2015) proposed a fully convolutional neural network FCN, which replaced the fully connected layer with a convolutional layer to achieve the first end-to-end image input–output method for pixel-level classification and improved the speed and accuracy of semantic segmentation. Ronneberger et al. (2015) proposed the symmetric structure of the fully convolutional neural network Unet, which uses jump links for feature fusion in the network and effectively alleviates the deep network degradation problem. Since then, fully convolutional neural networks have been extensively developed, and semantic segmentation is more often applied in building extraction tasks. Wang et al. (2018) propose a cascade coarse-to-fine network called CasNet, which focuses on regions that are difficult to make pixel-level labels. Obeso et al. (2017) propose a convolutional neural network to classify images of buildings using sparse features at the input of network in conjunction with primary color pixel values. Tang et al. (2019) proposed a random weighted averaging for the problem of overfitting in the results of Unet method. Zhou et al. (2022) proposed a novel efficient deep-wise spatial attention network (EDSANet), which uses dual attention extraction and attention feature refinement to aggregate multi-level semantics and enhance the accuracy of building extraction, especially for high spatial resolution imagery. Wang et al. (2022) combined the FPN structure with Unet++ so that the feature maps of each layer can be linked with the pyramid horizontally layer by layer to better extract the deep features of the image. Liu et al. (2021) proposed a context transfer network based on UNet (CT-UNet), which designed dense boundary blocks, spatial channel attention blocks, and improved loss functions. Fan et al. (2023) proposed a ResAt-UNet given the problem that the down-sampling of UNet makes it easy to lose context and detail information, attention mechanism, and residual module are added, which enhances the network depth, improves the fitting performance, and extracts small objects more accurately. The above studies show that Unet outperforms segmentation models such as FCN and ResNet in the building extraction task, and its unique symmetric structure is more helpful for the model to learn building features at different scales.

Many studies employ Unet as the backbone of the corresponding network. For example, He et al. (2022) proposed an E-Unet architecture combined with void convolution, which improved the extraction accuracy of buildings and improved the extraction edge corner ambiguity and detail loss. Yu et al. (2022) proposed an innovative Attention Gates U-Net (AGs-Unet), which can automatically learn diverse building structures from high-resolution remote sensing images. This is built upon the newly introduced attention gate module (AG) specifically for building extraction tasks. Qiu et al. (2023) proposed an improved network based on the UNet structure (Re-fine-UNet). The proposed Refine-UNet mainly consists of an encoder module, a decoder module, and a refined skip connection scheme. The refined skip connection scheme is composed of an atrous spatial convolutional pyramid pooling (ASPP) module and several improved depthwise separable convolution (IDSC) modules. Although these models obtain better performance of building extraction by replacing the original convolution layer of Unet, these methods bring more complexity to the models, such as more parameters for training, and suffer the problem of incomplete extraction of building contours in high-resolution remote sensing images.

To solve the problems, we propose an improved Unet network by integrating efficient convolution block attention (ECBA) and boundary detection to capture more comprehensive information of buildings. ECBA employs efficient channel attention as channel attention and the spatial attention of the convolutional block attention module (CBAM) as spatial attention. Channel information of the cross-channel joint feature map is achieved with the help of group convolution, and the plug-and-play feature of CBAM is retained to compose a lightweight attention module with more comprehensive extracted information. Rather than employing post-processing to optimize the building contours (Moghalles et al., 2022; Liu et al., 2020), a novel weighted loss function, i.e., a linear combination of dice loss and boundary loss, is designed to implement the boundary detection of building in an end-to-end method without changing the complexity of the method.

The main contributions of the study are as follows:

1) ECBA is proposed by sequentially combining dual attention mechanisms. ECBA could learn the specified features in both channel and space dimensions and focus on which information to emphasize or suppress.
2) A novel weighted loss function method is proposed to solve the unclear segmentation of building boundaries. The loss function is used to strengthen the segmentation of the building boundary in an end-to-end method.
3) Extensive experiments demonstrate that AMBDNet achieves superior recall, IoU, and pixel accuracy than traditional Unet and attention-based variants. This validates the effectiveness of combining ECBA with the boundary loss function for high-resolution building segmentation.

## 2 Proposed method

**Figure 1:**
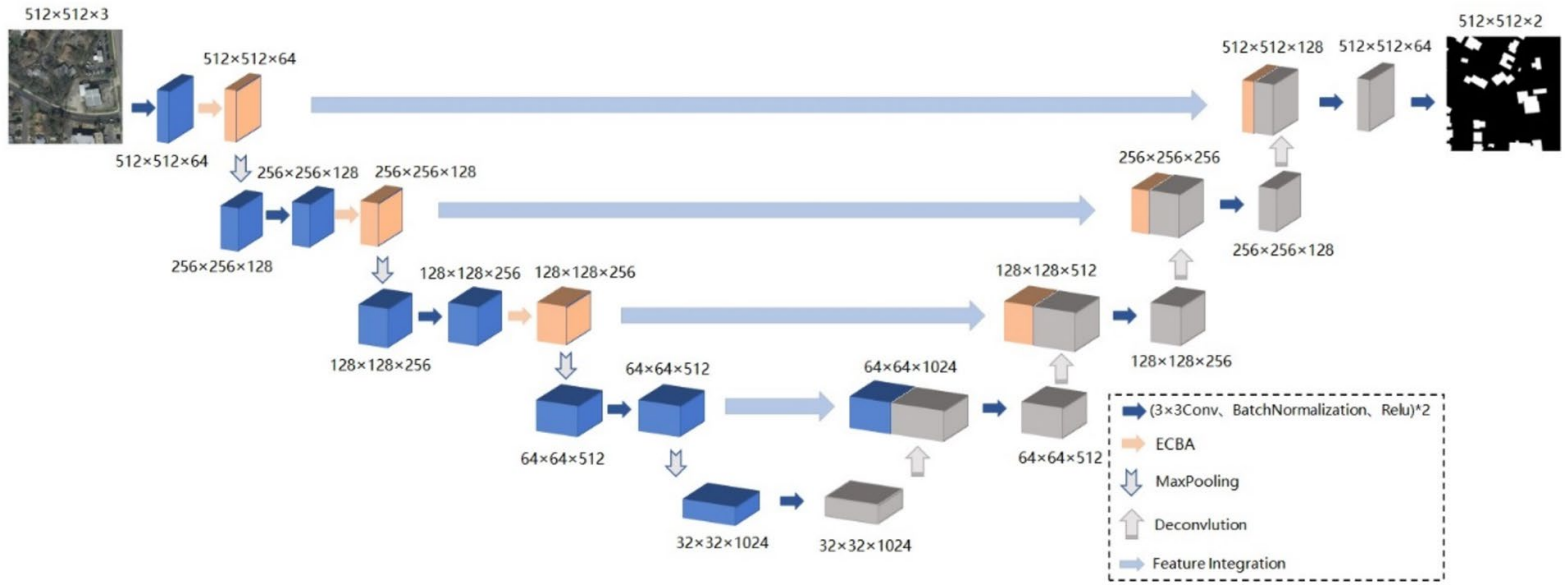
Unet_ECBA structure diagram.

The Unet network is one of the most excellent basic models for semantic segmentation, but the fixed structure of Unet and its simple network architecture cannot cope with the overly complex situations in high-resolution remote sensing images. To address the problem, AMBDNet is proposed by integrating a dual-attention mechanism module ECBA and a weighted loss function into the Unet network. First, the dual-attention mechanism ECBA could help the network focus on important building features and suppress unnecessary background features. Second, the weighted loss function could mitigate the incomplete identification of building boundaries due to shadow occlusion. Thus, the model could acquire sufficient contextual information in high-resolution image segmentation tasks and is prone to solve the problem of missed extraction and incomplete boundary recovery. The network structure is shown in Figure 1.

### 2.1 Backbone

**Figure 2:**
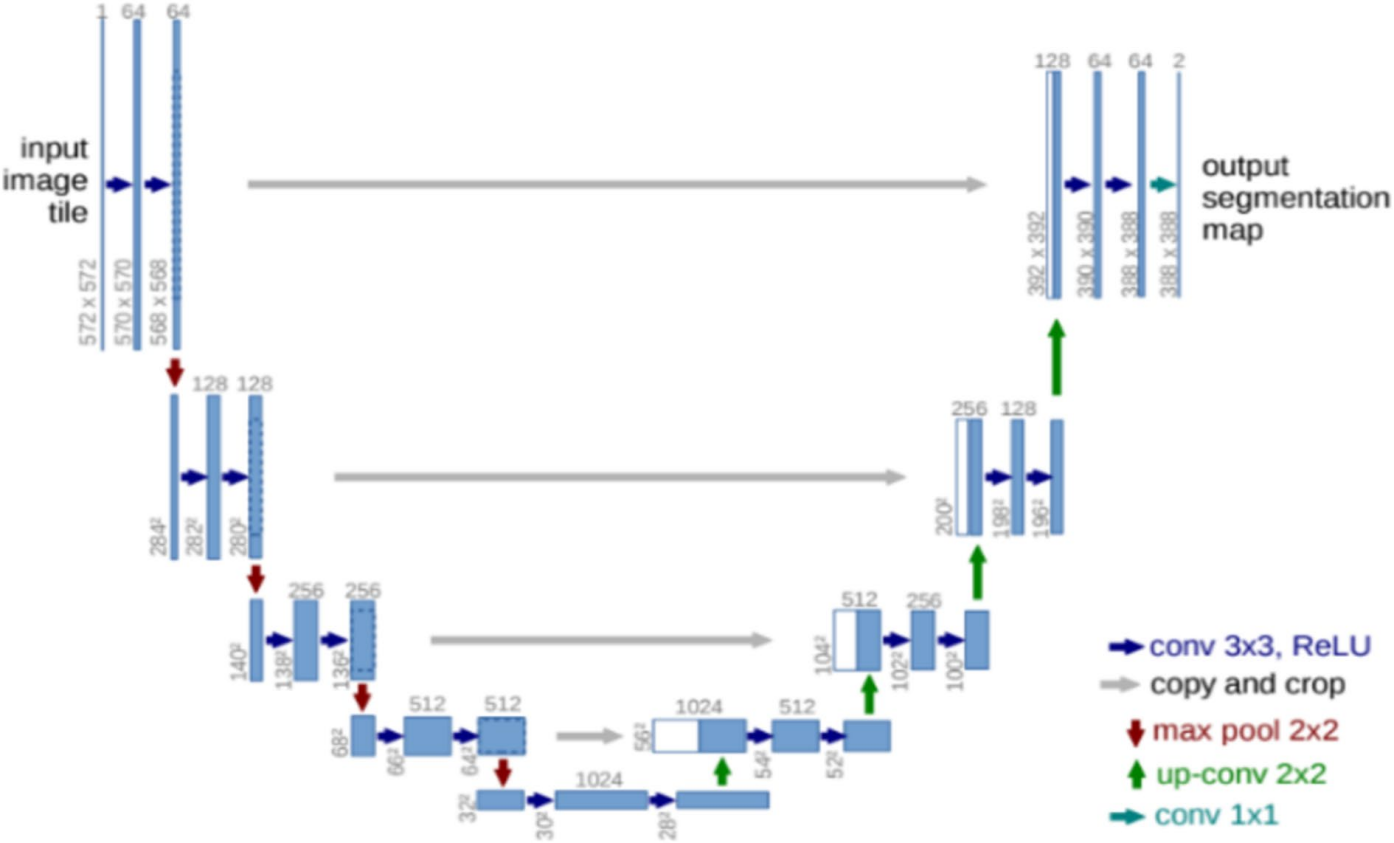
Unet structure.

Unet (Ronneberger et al., 2015) is one of the most basic classical segmentation networks, where the Unet structure is shown in Figure 2. Unet consists of the encoder network and the decoder network. The encoder network has 10 convolutional layers to extract the abstract feature information of the input image, and each group of two convolutional layers is bridged with a rectified linear unit (ReLU) and a max pooling operation. Four transposed convolutional layers are present in the decoder network to recover the image. Each transposed convolutional layer is connected to the corresponding layer in the downsampling stage, a concatenation with the correspondingly cropped feature map from the encoder network, and two convolutions, each followed by a ReLU function.

The proposed method AMBDNet integrates the dual-attention mechanism ECBA and the weighted loss function into the Unet network. The corresponding network architecture is shown in Table 1, where *N* is the number of repetitions of the module. In the encoder stage, a 3 × 3 convolution is used to encode the image. In the first three layers, the ECBA module is incorporated to enhance the extraction of the network model of building image features. To prevent overfitting, the ReLU activation function is bridged after each convolution operation, and the feature map is normalized after each convolution operation to prevent the gradient from disappearing during the training process. The decoder stage is completed by the upsampling operation of the de-convolution. Moreover, the feature map of the encoder stage is integrated by using two 3 × 3 convolutions. Finally, the number of feature map channels is compressed using 3 × 3 convolution to obtain the feature map of the original image size, and then, the classification probability map of each pixel is obtained by Softmax activation function.

**Table 1:** AMBDNet architecture.

Stage	Operator	*N*	Channels
Encoder	Conv3 × 3 Conv3 × 3 ECBA MaxPooling	3	64 128 256
	Conv3 × 3 Conv3 × 3 MaxPooling	1	512
	Conv3 × 3	2	1,024
Decoder	ConvTranspose3 × 3 Conv3 × 3 Conv3 × 3	4	512 256 128 64
	Conv3 × 3	2	2

### 2.2 Efficient convolution block attention module

**Figure 3:**
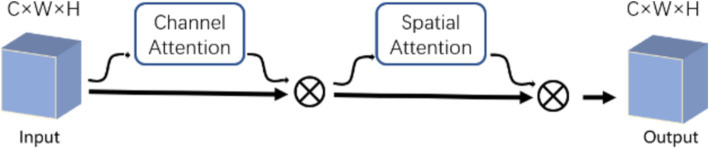
Diagram of ECBA module.

The ECBA module consists of a channel attention and a spatial attention. Inspired by CBAM (Woo et al., 2018), we also sequentially apply channel and spatial attention modules to learn ‘what’ and ‘where’ to attend in both modules, respectively. ECBA employs efficient convolution attention (ECA) (Wang et al., 2020) as the channel attention module followed by the original spatial attention module. The structure of the ECBA module is shown in Figure 3.

**Figure 4:**
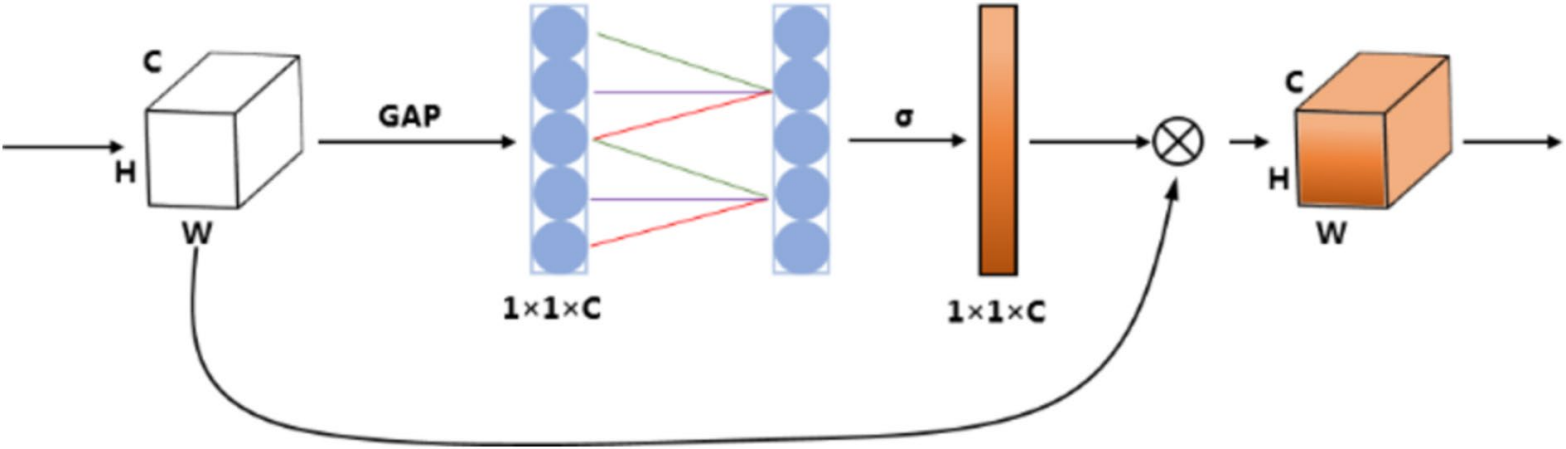
Diagram of the ECA module. GAP means Global Average Pooling.

The ECA module is employed as the channel attention, which could provide appropriate cross-channel interaction and avoid dimensionality reduction. The network is implemented by a fast 1D convolution of kernel size, where kernel size is proportional to channel dimension. In detail, the input feature map is processed by global average pooling to obtain the aggregated spatial information of the feature map; then, 1D convolution is performed followed by a Sigmoid function with the convolution kernel size adaptively selected to learn the channel attention; finally, the channel attention is multiplied with the input feature map to obtain the final feature map. The detailed mathematical functions are as follows:


$$M_{c}=\sigma \left(\mathrm{conv}1d\left(F_{avg}^{c}\right)\right)$$

$$F_{\mathrm{out}}^{c}=M_{c}\times F_{\mathrm{input}}$$

where c is the number of input feature map channels, $F_{avg}^{c}$ is the average pooled processed feature map, conv1d is the one-dimensional convolution, σ is the Sigmoid activation function, $M_{c}$ denotes the attention weight of the feature map on the channel, $F_{\mathrm{input}}$ is the input feature map, and $F_{\mathrm{out}}^{c}$ is the final output feature map. The corresponding diagram of the ECA module is shown in Figure 4.

**Figure 5:**
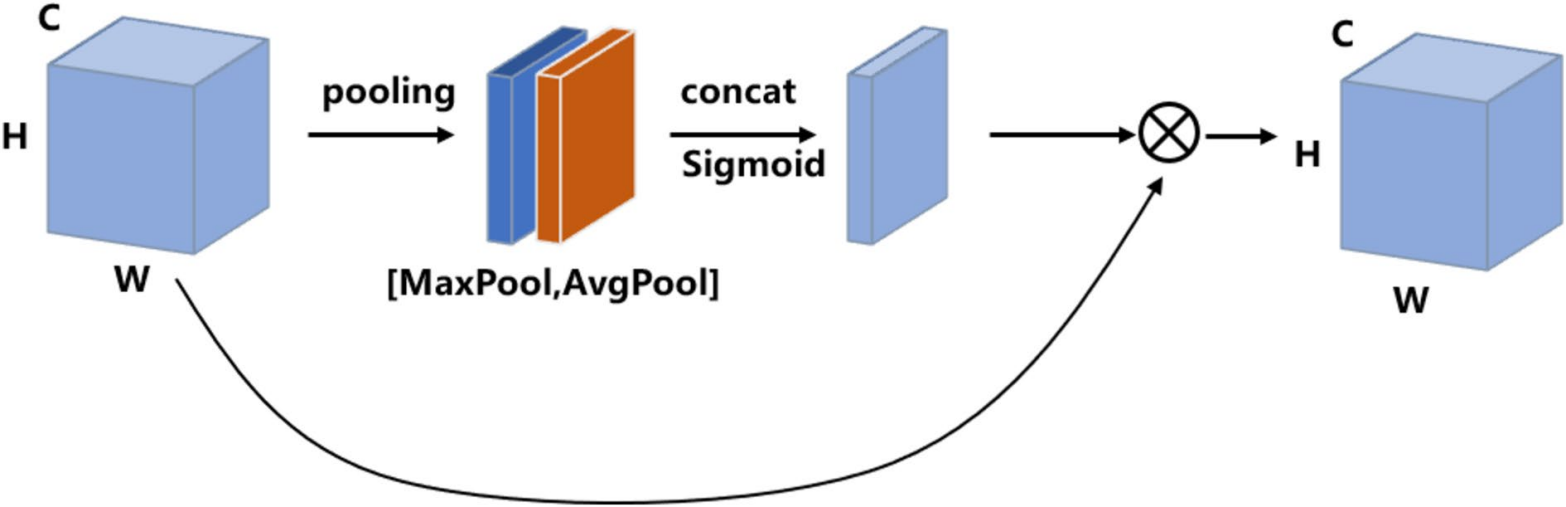
Diagram of the spatial attention module.

Spatial attention focuses on the inter-spatial relationship of features, which are used to capture the important spatial features. In detail, the input feature map is processed by average-pooling and max-pooling operations, respectively, then both are concatenated into a feature map in the channel dimension. The feature map is then passed to a convolution layer followed by the Sigmoid activation function. Finally, the final output feature map is obtained by multiplying the feature weights of spatial attention with the input feature map. The detailed mathematical functions are as follows:


$$M_{s}=\sigma \left(\mathrm{conv}2d^{7\times 7}\left(\left[F_{\text{max}};F_{avg}\right]\right)\right)$$

$$F_{\mathrm{out}}=M_{s}\times F_{\mathrm{input}}^{\prime }$$

where $F_{\text{max}}$ is the feature map generated by the max-pooling operation, $F_{avg}$ denotes the feature map processed by the average-pooling operation, $\mathrm{conv}2d^{7\times 7}$ denotes a two-dimensional convolution operation with a convolution kernel size of 7 × 7, σ is the Sigmoid function, and $M_{s}$ denotes the spatial attention. The spatial attention module is shown in Figure 5.

### 2.3 Loss function

The boundary segmentation of buildings is a difficult task in semantic segmentation. The fully convolutional neural network performs size reduction by the extracted building features in the upsampling phase but learns less information about the edges of buildings in the high perceptual field phase of the network, which leads to incomplete contours and distorted contours in the network extracted buildings.

To solve the problems, we design a weighted loss function combining dice loss and boundary loss (Kervadec et al., 2019), which controls the network model to learn more boundary features of the building. The weighted loss function is as follows:


$$\mathrm{Loss}={\mathrm{Loss}}_{d}\left(\mathrm{lab,pre}\right)+\alpha \times {\mathrm{Loss}}_{B}\left(\mathrm{lab,pre}\right)\text{,}$$

where ${\mathrm{Loss}}_{d}$ is the dice loss function, ${\mathrm{Loss}}_{B}$ is the boundary loss, lab is the image label, pre is the predicted image, and α is a weight parameter.

#### 2.3.1 Dice loss

Dice loss is a region-dependent loss function where the loss of a pixel point is not only correlated with the label and predicted value of that point, but also with the label and predicted value of other points, representing a region-dependent loss.


$${\mathrm{Loss}}_{d}=\frac{2\sum_{i}^{N}p_{i}g_{i}}{\sum_{i}^{N}p_{i}^{2}+\sum_{i}^{N}g_{i}^{2}}$$

where $p_{i}\in P$ is the binary segmentation of the predicted by the network and $g_{i}\in G$ is the ground truth segmentation. N is the total number of pixels. P and G can be 3D voxels or 2D pixels. Overall summary, it is the sum of two matrices that are bitwise multiplied, multiplied by 2, and divided by the bitwise squares of the two matrices.

#### 2.3.2 Boundary loss

Inspired by the study of Kervadec et al. (2019), we also employ boundary loss to train our model to deal with the unbalanced data problem. The boundary loss takes the form of a distance metric on the space of contours, not regions. The corresponding form can be approximated by


$${\mathrm{Loss}}_{B}\left(\theta \right)=\underset{\omega }{\int }\phi_{G}\left(q\right)s_{\theta }\left(q\right)d_{q}$$

where ω and q denote the spatial domain and a specific pixel within it. $\phi_{G}$ and $s_{\theta }$ denote the representation of the ground-truth region G and Softmax probability outputs of the network, respectively. In our experiment, the boundary loss is the sum of linear functions of the regional Softmax probability outputs of the network.

## 3 Experiments

### 3.1 Dataset

**Figure 6:**
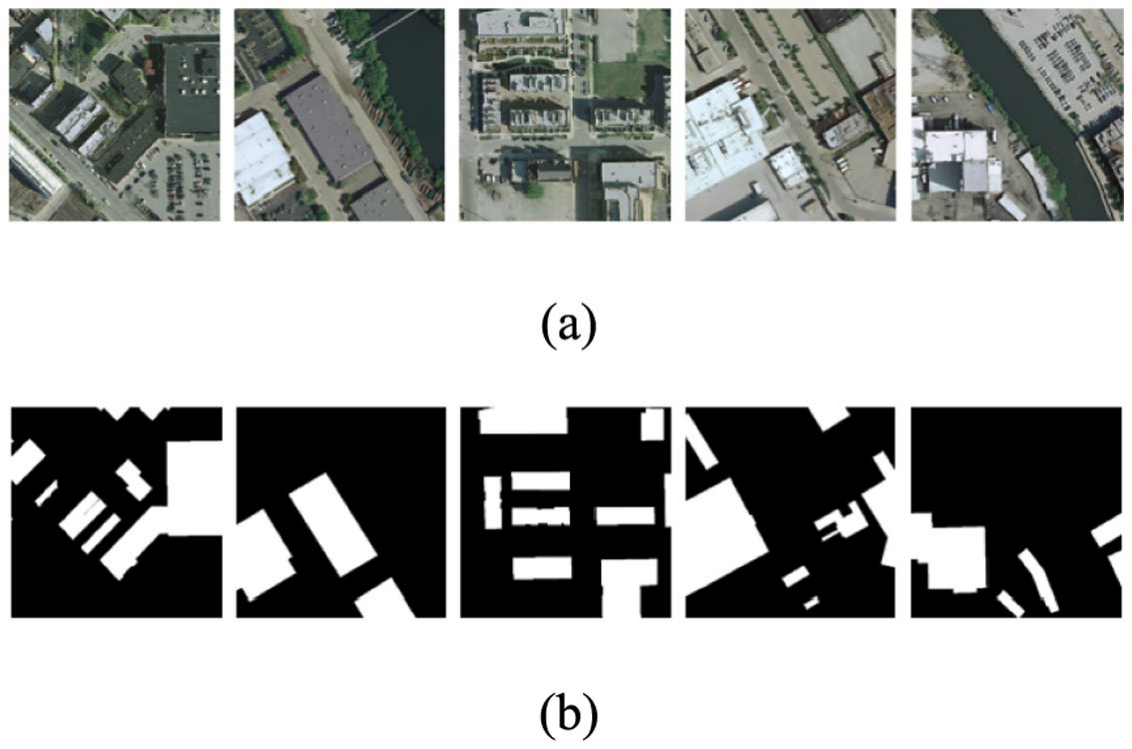
Examples of experimental data. Panel **(A)** represents images and **(B)** represents buildings and backgrounds with white and dark colors, respectively.

The Inria Aerial Image Labeling dataset (Maggiori et al., 2017) is selected for building extraction in our experiments. The size of each image in the dataset is 5,000*5,000 pixels, and the corresponding resolution is 0.3 m. Each image is a three-channel RGB image. The images are labeled with two categories, i.e., buildings and backgrounds. We select the large-scale images with more buildings as the experimental data. The large-scale images are divided into 4,372 patches of size 512×512 using a sliding window approach. We select 3,971 images as the training and validation sets and 401 images as test sets. Figure 6 depicts images and labels from a subset of this dataset, where the buildings are represented in white and the backgrounds are represented in black.

### 3.2 Experimental parameter setting

The data were normalized using the transformations with mean of 0.485, 0.456, and 0.406 and corresponding SD of 0.229, 0.224, and 0.225 for the RGB channels of the images, respectively.

For training, the input image size is 512*512, the batch size is set as 8, the epoch is set as 120, and the initial learning rate is 10^−5^. The gradient optimizer is the adaptive gradient descent algorithm (Kingma and Ba, 2014) and the equal interval learning rate decay strategy is selected to optimize the learning rate, which is determined to decay at 0.5 for every 10 training rounds. The attenuation factor is set as 0.5 because the factor can ensure the model has a good convergence speed with a small learning rate and the stable convergence of the model parameters to the optimal solution. For the hyperparameter *α* of the loss function, it is determined that the initial weight is 0.01 and increases by 0.001 for each round of experiments with repeated experiments. The purpose of such a setting is to make the model focus on the boundary features of the building in the later stage of training, and the central main features of the building are still the main focus in the early stage of training.

All programs run on a PC equipped with Ubuntu 16.04.4 operation system, Tesla M40 GPU with 24GB memory, python 3.7, PyTorch 1.9.0, and CUDA 10.2.

### 3.3 Evaluation metrics

Following existing studies, we use pixel accuracy (PA), intersection over union (IoU), mean intersection over union (MIoU), and Recall (R) to evaluate the performance of the models.

PA is the ratio of the number of correctly classified pixel points to the number of all pixel points, which indicates the accuracy of recognizing pixel objects. IoU is the intersection of the predicted labels and the ground-truth labels divided by the concatenation of the predicted labels and the ground-truth label. MIoU is the average IoU over all classes. R is the ratio of correctly classified pixels to the number of all pixels predicted to be in that category. The corresponding mathematical formulas are as follows:


$$PA=\frac{TP+TN}{TP+TN+FP+FN}$$

$$IoU=\frac{TP}{TP+FP+FN}$$

$$\mathrm{MIoU}=\frac{1}{2}\left({IoU}_{0}+{IoU}_{1}\right),{IoU}_{i}=\frac{{TP}_{i}}{{TP}_{i}+{FP}_{i}+{FN}_{i}},i=0,1$$

$$R=\frac{TP}{TP+FN}$$

The true positive (TP) class is denoted as the amount of accurately predicted building pixels. The false positive (FP) class represents the incorrectly estimated building pixel number. The true negative (TN) class is the correctly classified non-buildings pixels. The false negative (FN) is the number of misclassified buildings. These classes are calculated using a confusion matrix in Table 2.

**Table 2:** Confusion matrix.

		Actual performance	
		1	0
Predicted Performance	1	TP	FP
	0	FN	TN

### 3.4 Baselines

To verify the performance of the proposed model, we selected three methods as baseline methods. The details of the baseline can be found below.

(1) Unet (Ronneberger et al., 2015): the traditional Unet network with the cross-entropy loss function.
(2) Unet_ECBA: incorporating the ECBA attention module into the Unet network with the cross-entropy loss function. As an ablation study, we evaluated the impact of the ECBA attention module and the weighted boundary loss function.
(3) DeeplabV3 (Chen et al., 2017): incorporating atrous convolution with various rates to capture multi-scale context for semantic image segmentation.

## 4 Results and discussion

### 4.1 Experimental results

We compare Unet, Unet_ECBA, and DeeplabV3 with the proposed method AMBDNet on the Inria Aerial Building Dataset. The experimental results are shown in Table 3. According to the results, the proposed method achieves state-of-the-art performance in terms of MIoU. In detail, our method obtains 0.9046, 0.7797, and 0.9140 in terms of R, IoU, and PA for the building class.

**Table 3:** Evaluation results of different models on the building datasets.

Model	R		IoU		PA		MIoU
	Building	Others	Building	Others	Building	Others	
Unet	0.8724	0.9300	0.7543	0.9314	0.8971	0.9664	0.8428
Unet_ECBA	0.8902	0.9414	0.7685	0.9467	0.9067	0.9683	0.8576
DeeplabV3	0.8476	0.9070	0.6017	0.8772	0.7747	0.8633	0.7394
AMBDNet	0.9046	0.9743	0.7797	0.9481	0.9140	0.9713	0.8639

To further demonstrate the effectiveness of the proposed AMBDNet, ablation experiments were conducted. We evaluated the impact of the ECBA attention module and the weighted boundary loss function individually by creating two simplified versions of the model: (1) Unet: Unet_ECBA without ECBA and (2) Unet_ECBA: AMBDNet without the weighted boundary loss function.

Compared with the conventional network Unet, Unet_ECBA significantly surpasses its performance by 1.76% MIoU-score. In detail, Unet_ECBA obtains 2.04% *R*-score, 1.88% IoU-score, and 0.84 PA-score improvements in terms of the building class compared with Unet. These improvements are attributed to the fact that Unet_ECBA can leverage channel spatial attention modules to obtain the dependencies of feature maps.

Compared with Unet_ECBA, AMBDNet significantly surpasses its performance by 0.73% MIoU-score. In detail, AMBDNet obtains 1.61% *R*-score, 1.46% IoU-score, and 0.80 PA-score improvement in terms of the building class compared with Unet_ECBA. These improvements are attributed to the efficient convolution block being able to learn the important features from spatial and multi-channel dimensions by using the new weighted boundary loss function instead of the original cross-entropy loss function for better model training and learning in building boundary detection.

Compared with DeeplabV3, AMBDNet significantly surpasses its performance by 16.83% MIoU-score. In detail, AMBDNet obtains 6.72% *R*-score, 29.58% IoU-score, and 17.98 PA-score improvements in terms of the building class compared with DeeplabV3. These improvements are attributed to the combination of the Unet network and the efficient convolution block attention mechanism based on the weighted boundary loss function proposed in this study.

**Figure 7:**
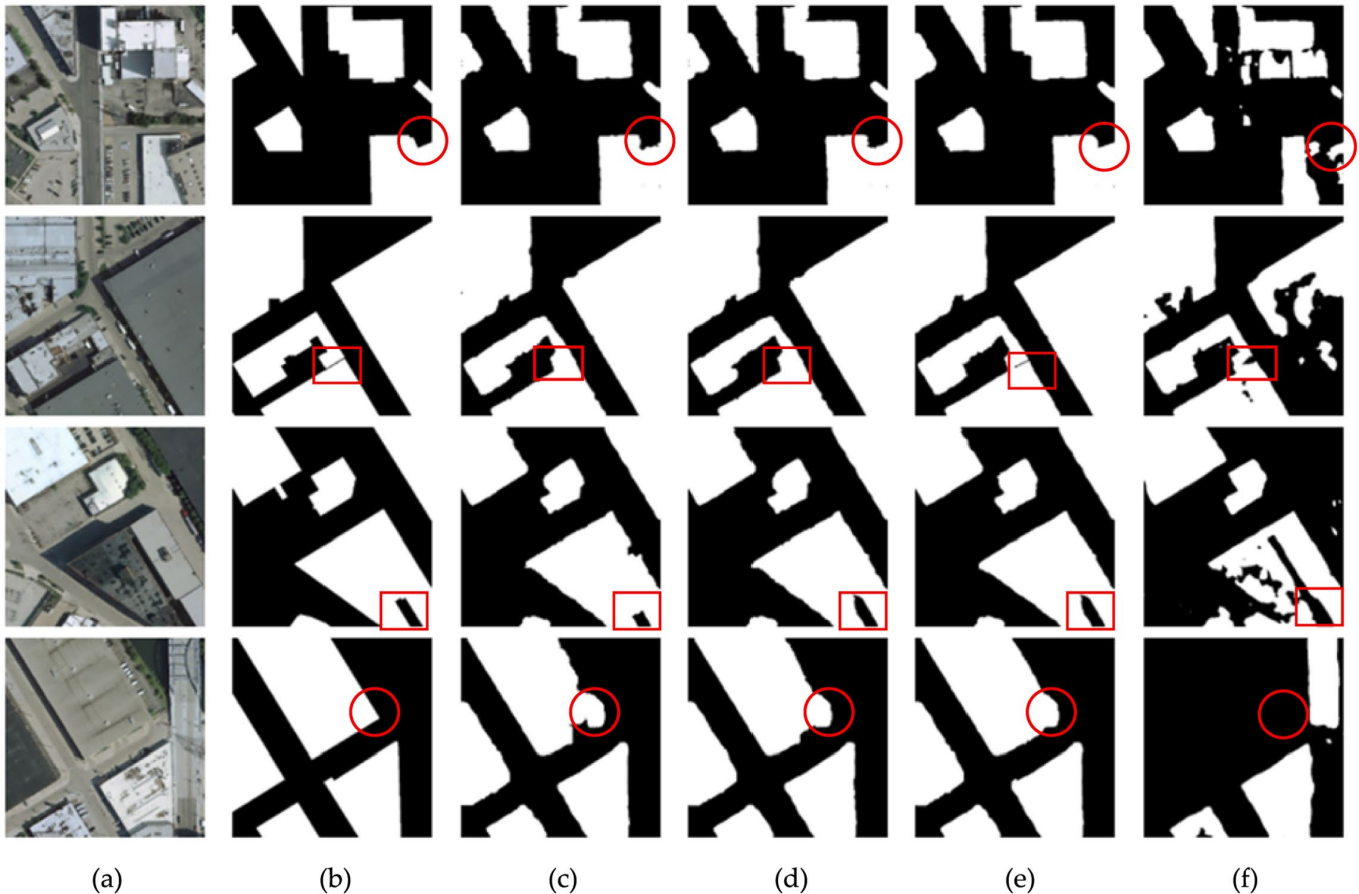
Comparison results on the building dataset. **(A)** Original images. **(B)** Corresponding labels. **(C)** Predicted maps of Unet. **(D)** Predicted maps of Unet_ECBA. **(E)** Predicted maps of AMBDNet. **(F)** Predicted maps of DeeplabV3.

Figure 7 compares the segmentation performance of different models on the building dataset. From the predicted maps, several key observations can be made: As seen in the red circular box in the first row, AMBDNet effectively captures detailed building edges compared to other models. This indicates that integration of AMBDNet of attention mechanisms enables it to better distinguish fine-grained boundary details, reducing misclassification around building edges. In the red rectangle of the second row, AMBDNet accurately segments building boundaries in shadowed areas, whereas other models such as Unet and Unet_ECBA fail to differentiate the boundaries properly. This suggests that the boundary loss function in AMBDNet plays a crucial role in enhancing the ability of the model to identify structures even under challenging lighting conditions. The circular box of the fourth row highlights regions with similar pixel values between buildings and backgrounds. While Unet and other models struggle with misclassification in these regions, AMBDNet successfully distinguishes the building features. This demonstrates that attention mechanism of AMBDNet is effective in reducing confusion between similar pixel intensities, leading to more accurate segmentation results.

In the comparison between Unet, Unet_ECBA, and AMBDNet, Figure 7 reveals that Unet_ECBA and AMBDNet can capture more relevant building pixels, particularly in the fourth row. This indicates that both models are better at extracting building features than the original Unet. Specifically, the red rectangles in the third row highlight areas where Unet_ECBA and AMBDNet successfully distinguish the shaded regions within the central cavity of the building, whereas Unet struggles to classify these regions accurately. This improvement can be attributed to the ECBA attention mechanism, which enhances the robustness of the model by reducing interference from non-building features.

Additionally, AMBDNet demonstrates superior edge detail extraction than Unet_ECBA, as evidenced by the clearer building boundaries in the fourth row of Figure 7. This is a result of the weighted boundary loss function, which enables AMBDNet to more accurately segment building boundaries, ensuring complete extraction even in challenging shadowed regions. Therefore, the observations from Figure 7 underscore the advantages of our model in capturing intricate building edge details.

Table 4 further supports these findings by showing the tradeoff between model complexity and performance. While AMBDNet marginally increases the training time compared to Unet and Unet_ECBA, it achieves significant improvements in segmentation accuracy without a substantial rise in model complexity. This balance between efficiency and effectiveness indicates that AMBDNet is capable of achieving enhanced segmentation performance without incurring significant computational costs.

**Table 4:** Number of parameters in different models.

Model	Parameters/MB	Time (epoch)/s
Unet	132.3	1842.41
Unet_ECBA	132.3	1850.87
AMBDNet	132.3	1885.93

### 4.2 Attention mechanisms in the upsampling and downsampling stages

The proposed method AMBDNet integrates the ECBA attention in all upsampling stages and no attention mechanism in all downsampling stages, thus achieving better segmentation performance. To further verify the fact that attentions works in the upsampling stages, we conduct more experiments in that attentions are incorporated into both the upsampling and downsampling stages. Here, four-layer and five-layer attention mechanism fusion are incorporated into the downsampling stages as AMBDNet_4 and AMBDNet_5, respectively. The evaluation results of different attentions on the building datasets are shown in Table 5.

**Table 5:** Results of the evaluation of the incorporation of additional layers of attention mechanisms in the downsampling stages on the reference dataset.

Model	Recall		IOU		PA		MIOU
	Building	Others	Building	Others	Building	Others	
AMBDNet	0.9046	0.9743	0.7797	0.9481	0.9140	0.9713	0.8639
AMBDNet_4	0.8910	0.9695	0.7735	0.9453	0.9085	0.9692	0.8592
AMBDNet_5	0.8936	0.9671	0.7719	0.9402	0.9043	0.9657	0.8560

We could observe that AMBDNet_4 and AMBDNet_5 achieve poor segmentation performance on the building dataset by integrating the excessive addition of the attention mechanisms in the downsampling stages. Attention mechanisms in downsampling stages focus on the important features map while the boundary loss function focuses on the building boundary. Thus, the conflict between the attention mechanism and the boundary loss function in the high perceptual field stage of the network may result in poor segmentation performance.

**Figure 8:**
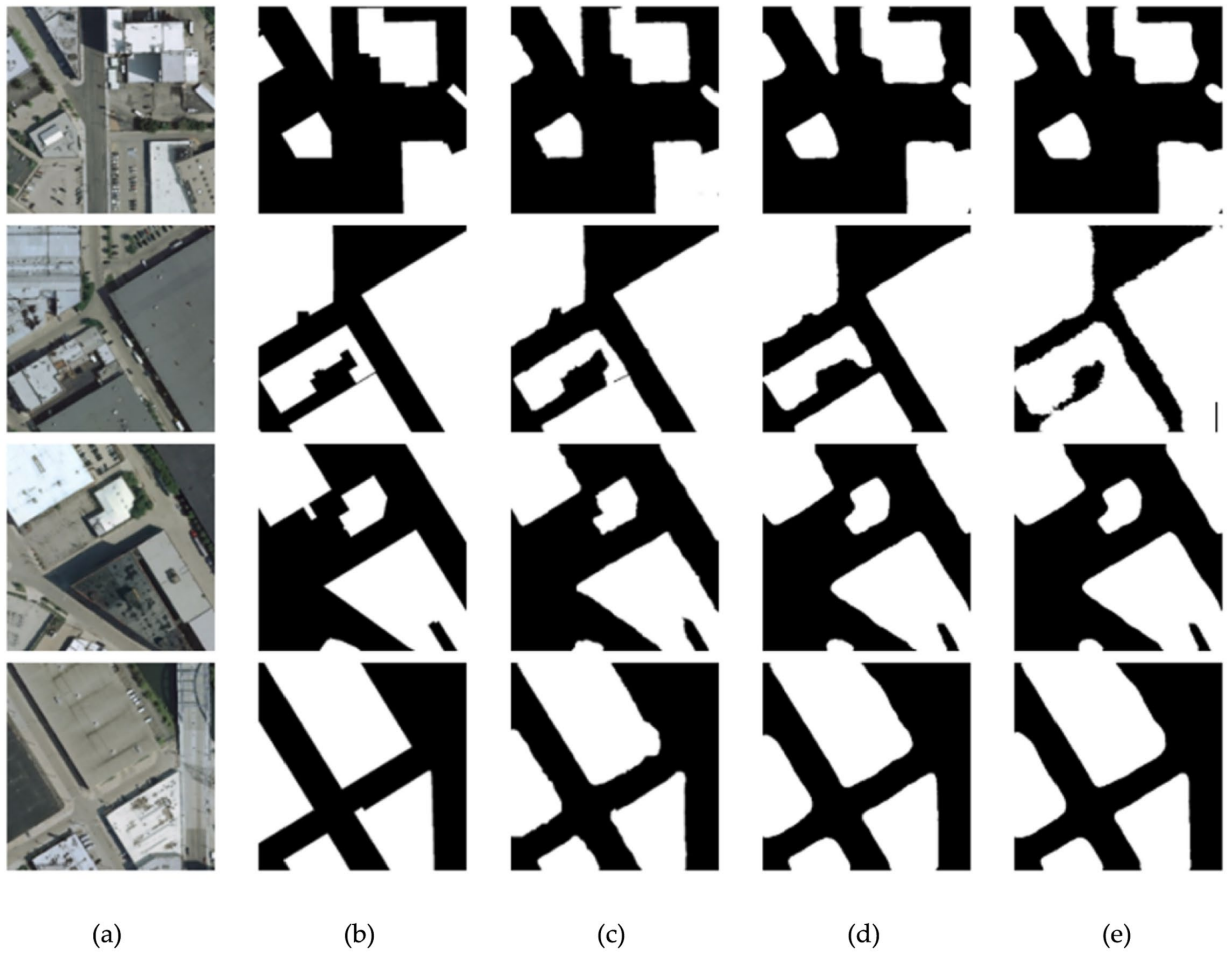
Comparison results of different attention mechanisms on the building dataset. **(A)** Original images. **(B)** Corresponding labels. **(C)** Predicted maps of AMBDNet. **(D)** Predicted maps of AMBDNet_4. **(E)** Predicted maps of AMBDNet_5.

Meanwhile, the predicted maps of different models with attention mechanisms in the upsampling and downsampling stages on the building dataset are shown in Figure 8. We could observe that as the attention mechanism increases in the downsampling stage, it instead leads to more generalized image predictions, with the building boundary segmentation lines showing rounding and poor results at the corners of the buildings, presenting worse results overall on the buildings.

### 4.3 Limitations of the research and future study

Unet and DeepLabV3 have been demonstrated to achieve better performance in tasks such as building segmentation and medical image segmentation. Thus, we incorporate the ECBA attention module into Unet to demonstrate the effectiveness of ECBA and the weighted boundary loss function. Despite these achievements, the proposed model has some limitations. First, the comparison between ECBA and different attention mechanisms is lacking and the fusion between ECBA and other baseline methods is needed to explore. Second, the model exhibits relatively high computational complexity and training time, which can hinder real-time applications. Additionally, the model is not as lightweight as desired for deployment on devices with limited computational resources. In future study, we would explore methods to reduce the complexity of model, such as pruning techniques or more efficient attention mechanisms, to strike a better balance between accuracy and speed. Moreover, we would strengthen the generalizability and robustness of the model on diverse datasets.

## 5 Conclusion

To address the challenges in accurately extracting buildings from high-resolution remote sensing images, our study proposed improvements to the Unet model by integrating an ECBA attention module and a weighted boundary loss function. The ECBA module, which combines channel and spatial attention, effectively addresses issues of missing and incorrect extractions of buildings and incomplete building contours that are common in traditional Unet models. This enhancement enables our model to better capture fine-grained features, leading to a significant improvement in segmentation quality. For addressing the problem of incomplete segmentation of building boundaries, particularly in shadowed areas or irregular boundaries, we replaced the traditional cross-entropy loss function with a weighted boundary loss function. This change allowed the model to better preserve boundary integrity during backpropagation, resulting in more accurate boundary delineation. The effectiveness of these methodological improvements is evident from the results: the proposed model achieved a recall rate of 0.9046, an intersection over union (IoU) of 0.7797, a pixel accuracy of 0.9140, and a mean intersection over union (MIoU) of 0.8639. These metrics highlight the ability of the model to outperform traditional approaches in both precision and boundary recognition. The enhanced performance of the model in shadowed and complex regions makes it suitable for applications in geographic information systems (GIS) and automated mapping technologies.

## Data Availability

Publicly available datasets were analyzed in this study. This data can be found at: https://project.inria.fr/aerialimagelabeling/.

## References

[ref1] Aytekın Ö. Erener A. Ulusoy İ. Düzgün Ş. (2012). Unsupervised building detection in complex urban environments from multispectral satellite imagery. Int. J. Remote Sens. 33, 2152–2177. doi: 10.1080/01431161.2011.606852

[ref2] Chen L. C. Papandreou G. Schroff F. Adam H. Rethinking atrous convolution for semantic image segmentation. [Epubh ahead of preprint]. doi: 10.48550/arXiv.1706.05587. (2017), PMID: .39713797

[ref3] Fan Z. Liu Y. Xia M. Hou J. Yan F. Zang Q. (2023). ResAt-UNet: a U-shaped network using ResNet and attention module for image segmentation of urban buildings. IEEE J. Top. Appl. Earth Observat. Remote Sens. 16, 2094–2111. doi: 10.1109/JSTARS.2023.3238720

[ref4] He Z. Ding H. An B. (2022). The cavity convolution E-Unet algorithm for building extraction from high-resolution remote sensing image. J. Surv. Map. 51, 457–467. doi: 10.11947/j.AGCS.2022.20200601

[ref5] Izadi M. Saeedi P. (2010). Automatic building detection in aerial images using a hierarchical feature based image segmentation.2010 20th international conference on pattern recognition. Istanbul, Turkey: IEEE, 472–475.

[ref6] Kervadec H. Bouchtiba J. Desrosiers C. Granger E. Dotz J. Ayed I. B. Boundary loss for highly unbalanced segmentation. International conference on medical imaging with deep learning. PMLR, (2019): 285–296. doi: 10.1016/j.media.2020.101851

[ref7] Kingma D. Ba J. (2014). Adam: a method for stochastic optimization. Computer Science. doi: 10.48550/arXiv.1412.6980

[ref8] Lin C. Nevatia R. (1998). Building detection and description from a single intensity image. Comput. Vis. Image Underst. 72, 101–121. doi: 10.1006/cviu.1998.0724

[ref9] Liu D. Han L. Han X. (2016). Research on high resolution remote sensing image classification based on deep learning. J. Opt. 36, 306–314. doi: 10.3788/AOS201636.0428001

[ref10] Liu K. Ma H. Ma H. Cai Z. Zhang L. (2020). Building extraction from airborne LiDAR data based on min-cut and improved post-processing. Remote Sens. 12:2849. doi: 10.3390/rs12172849

[ref11] Liu S. Ye H. Jin K. Cheng H. (2021). CT-UNet: context-transfer-UNet for building segmentation in remote sensing images. Neural. Process. Lett. 53, 4257–4277. doi: 10.1007/s11063-021-10592-w

[ref12] Long J. Shelhamer E. Darrell T. (2015). Fully convolutional networks for semantic segmentation. Proc. IEEE Conf. Comput. Vis. Pattern Recognit. 39, 640–651. 10.1109/TPAMI.2016.2572683 27244717

[ref13] Maggiori E Tarabalka Y Charpiat G Alliez P. Can semantic labeling methods generalize to any city? The inria aerial image labeling benchmark. (2017) IEEE International Geoscience and Remote Sensing Symposium (IGARSS). IEEE. 3226–3229.

[ref14] Meedeniya D. A. Jayanetti J. M. Dilini M. D. N. Wickramapala M. H. Madushanka J. H. (2020). “Land-use classification with integrated data” in Machine vision inspection systems: image processing, concepts, methodologies and applications, vol. 1, Eds. Muthukumaran, M., Soumya, R. N., Surya, N. P., Prasant, K. P., and Nittaya, M., (Scrivener Publishing LLC), 1–36.

[ref15] Moghalles K. Li H. C. al-Huda Z. Raza A. Malik A. (2022). Weakly supervised building semantic segmentation via superpixel-CRF with initial deep seeds guiding. IET Image Process. 16, 3258–3267. doi: 10.1049/ipr2.12558

[ref16] Obeso A. M. Benois-Pineau J. Acosta A. Á. R. Vázquez M. S. G. (2017). Architectural style classification of mexican historical buildings using deep convolutional neural networks and sparse features. J Electron Imaging 26, –011016. doi: 10.1117/1.JEI.26.1.011016, PMID: 39697822

[ref17] Qiu W. Gu L. Gao F. Jiang T. (2023). Building extraction from very high-resolution remote sensing images using refine-UNet. IEEE Geosci. Remote Sens. Lett. 20, 1–5. doi: 10.1109/LGRS.2023.3243609, PMID: 39573497

[ref18] Ronneberger O. Fischer P. Brox T.. U-net: convolutional networks for biomedical image segmentation. Medical image computing and computer-assisted intervention–MICCAI 2015. 18th International Conference, Munich, Germany, October 5-9, 2015, proceedings, part III 18. Springer International Publishing, (2015): 234–241.

[ref19] Tang P. Liang Q. Yan X. Xiang S. Sun W. Zhang D.. (2019). Efficient skin lesion segmentation using separable-Unet with stochastic weight averaging. Comput. Methods Prog. Biomed. 178, 289–301. doi: 10.1016/j.cmpb.2019.07.005, PMID: 31416556

[ref20] Wang Z. Deng Z. Wang S. (2018). CasNet: a cascade coarse-to-fine network for semantic segmentation. Tsinghua Sci. Technol. 24, 207–215. doi: 10.26599/TST.2018.9010044

[ref21] Wang K. Fan X. Wang Q.. FPB-UNet++: semantic segmentation for remote sensing images of reservoir area via improved UNet++ with FPN. 2022 the 6th International Conference on Innovation in Artificial Intelligence (ICIAI). (2022): 100–104.

[ref22] Wang Q. Wu B. Zhu P. Li P. Zuo W. Hu Q. ECA-net: efficient channel attention for deep convolutional neural networks. Proceedings of the IEEE/CVF Conference on Computer Vision and Pattern Recognition. (2020): 11534–11542.

[ref23] Wegne J. D. Soergel U. Rosenhahn B. (2011). Segment-based building detection with conditional random fields.2011 joint urban remote sensing event. Munich, Germany: IEEE, 205–208.

[ref24] Woo S. Park J. Lee J. Y. Kweon I. S. Cbam: convolutional block attention module. Proceedings of the European conference on computer vision (ECCV). (2018): 3–19.

[ref25] Yu M. Chen X. Zhang W. Liu Y. (2022). AGs-Unet: building extraction model for high resolution remote sensing images based on attention gates U network. Sensors 22:2932. doi: 10.3390/s22082932, PMID: 35458917 PMC9031445

[ref26] Zhou J. Liu Y. Nie G. Cheng H. Yang X. Chen X.. (2022). Building extraction and floor area estimation at the village level in rural China via a comprehensive method integrating UAV photogrammetry and the novel EDSANet. Remote Sens. 14:5175. doi: 10.3390/rs14205175

